# The Effect of $1, $5 and $10 Stakes in an Online Dictator Game

**DOI:** 10.1371/journal.pone.0073131

**Published:** 2013-08-12

**Authors:** Nichola J. Raihani, Ruth Mace, Shakti Lamba

**Affiliations:** 1 Department of Genetics, Evolution and Environment, University College London, London, United Kingdom; 2 Department of Anthropology, University College London, London, United Kingdom; 3 Centre for Ecology and Conservation, University of Exeter Cornwall Campus, Cornwall, United Kingdom; University of Debrecen, Hungary

## Abstract

The decision rules underpinning human cooperative behaviour are often investigated under laboratory conditions using monetary incentives. A major concern with this approach is that stake size may bias subjects’ decisions. This concern is particularly acute in online studies, where stakes are often far lower than those used in laboratory or field settings. We address this concern by conducting a Dictator Game using Amazon Mechanical Turk. In this two-player game, one player (the dictator) determines the division of an endowment between himself and the other player. We recruited subjects from India and the USA to play an online Dictator Game. Dictators received endowments of $1, $5 or $10. We collected two batches of data over two consecutive years. We found that players from India were less generous when playing with a $10 stake. By contrast, the effect of stake size among players from the USA was very small. This study indicates that the effects of stake size on decision making in economic games may vary across populations.

## Introduction

Researchers interested in the evolution of cooperative behaviour in humans often conduct economic games under controlled laboratory settings, predominantly using Western undergraduates as the subjects in these experiments. Despite providing huge insights into the factors underpinning social behaviour in humans, this approach has been criticised on the grounds that Western undergraduate samples are unlikely to be representative of humans as a whole [1] since human behaviour is expected to vary across populations [2,3]. Partly in response to this critique, some researchers have now turned to using online methods to investigate variation in human social behaviour. In particular, online labour markets, such as Amazon’s Mechanical Turk (AMT), allow researchers to recruit large numbers of subjects from a diverse range of backgrounds and from different countries at relatively small costs [4,5]. However, a key issue with the proliferation of studies using online labour markets is whether the small stakes that are typically used in online economic games systematically affect the decisions made by players in these games. We investigate this issue here.

One of the simplest games for investigating variation in prosocial behaviour is the Dictator Game [6,7]. In this two-player game, one individual (the dictator) is endowed with a sum of money and is told he may give some, all or none of this endowment to a second player (the receiver). The receiver must accept any offer made by the dictator. The income-maximising strategy for dictators is to keep the entire endowment and offer nothing to the receiver. However, this strategy is not observed empirically. Instead, meta-analysis based on laboratory studies using predominantly Western undergraduate samples has shown that on average dictators tend to transfer around 28% of the endowment to receivers [8]. Previous laboratory studies have shown that dictator donations are unaffected by endowment (or stake) size. For example, Forsythe et al. [9] found no effect of increasing stake size from $5 to $10 on dictator transfers, while Carpenter et al. [10] compared dictator transfers under $10 and $100 stakes and also found no difference in relative allocation to receivers. However, the effect of stake size in the online labour market AMT has been less well explored. In a recent study, Amir et al. [11] found that AMT dictators were significantly less generous when playing for real money (a $1 stake) than when no stakes were involved. This study did not assess whether varying the stake size in the online environment also affected dictator donations. This is important because most studies using AMT tend to use stakes that are much smaller than those used in the physical laboratory: standard stakes in AMT are $1 or less [11], while standard stakes in laboratory Dictator Games are usually $10 [8]. Moreover, evidence suggests that people’s responses to zero rewards are qualitatively different to responses to small, positive rewards [12,13], indicating that non-linearity in responses between zero-stakes and small-stakes conditions might also exist in economic games. In this study we use AMT to run a Dictator Game with stake sizes of $1, $5 and $10 respectively. We test whether stake size affects dictator donations after controlling for several demographic variables.

## Material & Methods

This research was approved by the University College London ethics board project number 3720/001. Subjects were informed that they would be taking part in an online experiment where they could earn money. All subjects remained anonymous so informed consent about the use of personal data was deemed unnecessary and was therefore waived by the University College London ethics board. Two batches of data were collected for this study were collected using the online labour market Amazon Mechanical Turk (www.mturk.com) in two batches, in March 2012 and March 2013. In 2012, 230 AMT workers were recruited and in 2013, 944 AMT workers were recruited. We restricted the game to workers from India (n=584) and the USA (n=590) since the majority of workers on AMT are from these countries [14]. Of the 1174 workers recruited, we assigned 587 workers to the role dictator (though the more neutral term 'decider' was used in the instructions) while the remaining 587 workers were assigned the role 'receiver'. We did not analyse any of the data submitted by receivers since they have no active role to play in the division of the endowment between dictator and receiver. Both workers were given written instructions about the game in English (see electronic supplementary material S1) and were required to answer two comprehension questions correctly to be eligible for the game. AMT workers are identified by a unique 14-digit code rather than their names. Workers were told that their ID would not be revealed to their partner in the game, thus ensuring anonymity. All participants answered a set of questions that provided demographic information (see electronic supplementary information S1 for further details). Of particular interest were age, gender and whether the participant had any children. The mean ages of our Indian and U.S.A. participants, as well as sample sizes associated with the different variables used in our analyses are given in Table 1. Stake size was balanced across years such that a similar proportion of participants each year received $1, $5, and $10 stakes respectively.

**Table 1:** Information on mean values ± SE (where appropriate) and sample sizes for the explanatory terms used in the statistical models.

**Parameter**	**India** (n = 282)	**USA** (n = 292)
Age	Mean = 28.9 ± 0.5	Mean = 28.4 ± 0.5
	Range = 18 - 65	Range = 17 - 65
Gender (n)	Females = 94	Females = 101
	Males = 188	Males = 191
Children (n)	No = 183	No = 234
	Yes = 99	Yes = 58
Stake size (n)	$1 = 95	$1 = 97
	$5 = 93	$5 = 99
	$10 = 94	$10 = 96
Year (n)	2012 = 57	2012 = 58
	2013 = 225	2013 = 234

Data were analysed using R version 3.0.1 [15]. We assessed whether stake size had a significant effect on dictator donations, while controlling for the demographic variables above. Dictator donation was calculated by dividing the amount transferred to the receiver by the stake ($1, $5, or $10). This value was arcsine-square root transformed and set as the response term in a linear model (LM, R function lm) with normal error distribution. We analysed the Indian dataset separately from the US dataset since, in this study, we did not have data from a sufficient number of countries to look at cross-cultural variation in dictator behaviour in any meaningful way.

**Table 2:** The top models (models within 2AICc units of the best model), with AICc values and Akaike weights (*w^i^*).

**Country**	**Model Rank**	**Parameters**	**df**	**AICc**	***w*^*i*^**
**India**	**1**	**Children + Stake + Year**	**6**	**82.0**	**0.40**
	2	Stake + Year	5	83.2	0.22
	3	Age + Stake + Year	6	83.4	0.20
	4	Children + Gender+ Stake + Year	7	83.7	0.17
**USA**	**1**	**Gender**	**3**	**194.2**	**0.51**
	2	Gender + Stake	5	195.2	0.30
	3	Age + Gender	4	196.2	0.19

The best model for each country is highlighted.

We created two general linear models (one for India, n = 282; one for the USA, n = 292) with the transformed value of dictator donation set as the response term. We used an information theoretic approach with model averaging as described in [16]. Under an information-theoretic approach, a series of candidate models are generated, with each model representing a biological hypothesis. Rather than testing a null hypothesis, the relative degree of support for each model from the candidate set is calculated [17]. By comparing different models, it is possible to determine the relative importance of different explanatory terms. For both India and the USA, a global model was first specified which included the following terms: dictator age, gender, stake size ($1, $5, or $10), whether the dictator had any children or not and the year that the data were collected (2012/2013). We also included possible two-way interactions between stake size and the other explanatory variables. Following the specification of the global model, the input variables were standardized according to [18]. Standardizing input variables allows the relative strength of parameter estimates to be interpreted. We used the package MuMIn [19] to derive and compare submodels from this initial global model (see 16 for all details). Models were compared to one another using Akaike’s Information Criterion corrected for small sample sizes (AICc) [20]. A subset of 'top models' were defined by taking the best model (the model with the lowest AICc value) and any models within 2AICc units of the best model (following [16,17]). Using this subset of models, we computed the average parameter estimates for each term included in the subset of models, as well as the relative importance of the term. Importance is calculated by summing the Akaike weights of all models where the term in question is included in the model. Akaike weights represent the probability of a given model being the true model (compared to other candidate models in the set) [17]. Importance can therefore be thought of as the probability that the term in question is a component of the best model [21]. In the results section, we only present the parameter estimates from the top models (those that were within 2 AICc units of the best model).

## Results

### INDIA

**Figure 1:**
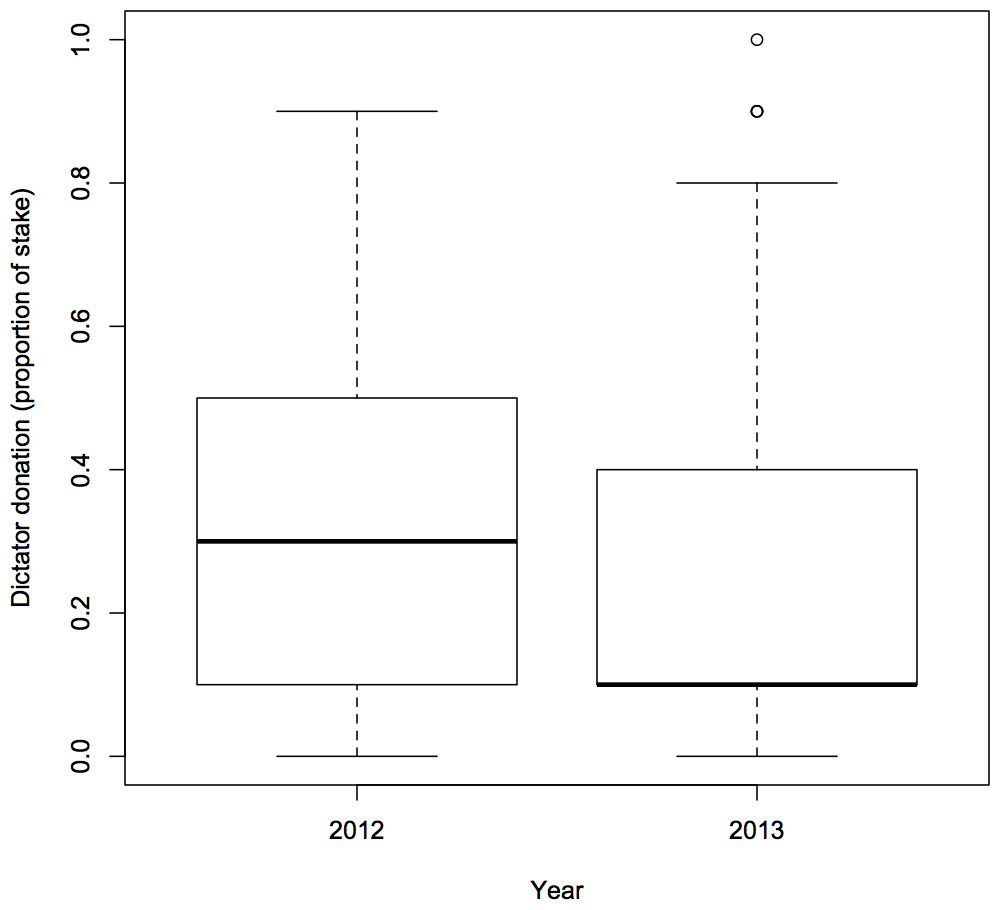
Boxplot of dictator donations (proportion of endowment given to receiver) according to year generated from raw data from Indian players. Solid lines represent medians while the upper and lower boundaries of the box are the upper and lower quartiles of the data. The highest and lowest values in the data (excluding outliers) are indicated by the bars extending from the boxes. Circles represent outliers.

**Figure 2:**
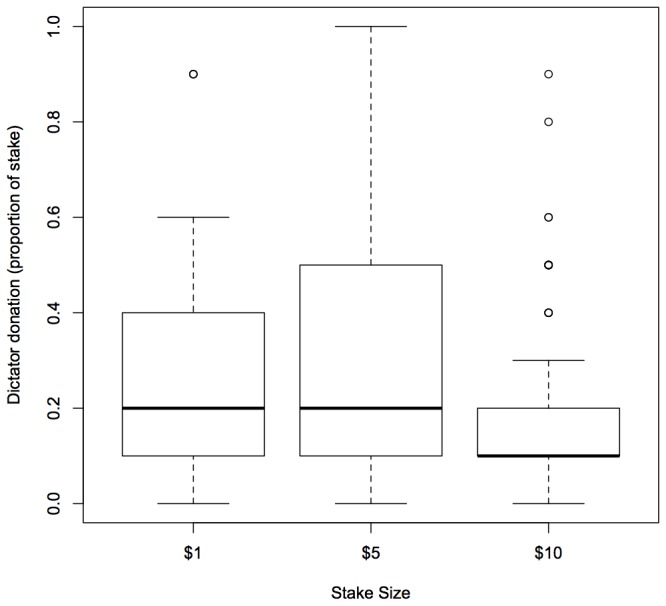
Boxplot of dictator donations (proportion of endowment given to receiver) according to stake size for Indian players. Solid lines represent medians while the upper and lower boundaries of the box are the upper and lower quartiles of the data. The highest and lowest values in the data (excluding outliers) are indicated by the bars extending from the boxes. Circles represent outliers.

For subjects from India, we found three models which were within 2AICc units of the best model (Table 2). Year and stake size were components of all the top models (Table 2). Our Indian players seemed to be less generous in the second year of the study (Figure 1) and also less generous when playing with the $10, as opposed to the $1 or $5, stake (Figure 2). However, there was no difference in donations between the $1 and $5 stakes. Players with children seemed to be more generous than players without children (importance = 0.57), although the confidence intervals for this term span zero meaning that this effect was quite weak. Other component variables included in the four top models were age (importance = 0.20) and gender (importance = 0.17). Again, for both these terms, the confidence intervals spanned zero meaning that they were unlikely to affect dictator donations (Table 3).

**Table 3:** Estimates, unconditional standard errors, confidence intervals and relative importance for parameters included in the top models for players from India.

**Parameter**	**Estimate**	**Unconditional SE**	**Confidence Interval**	**Relative Importance**
Intercept	0.49	0.03	(0.43, 0.55)	
Stake				1.00
$1	0.00	0.00	(0.00, 0.00)	
$5	-0.01	0.04	(-0.09, 0.07)	
$10	-0.12	0.04	(-0.20, -0.04)	
Year (2012/2013)	-0.14	0.04	(-0.22, -0.06)	1.00
Children (N/Y)	0.06	0.04	(-0.01, 0.13)	0.57
Age	0.05	0.03	(-0.02, 0.11)	0.20
Gender (F / M)	0.02	0.04	(-0.05, 0.10)	0.17

Effect sizes have been standardized on two SD following [18] and standard errors are unconditional, meaning that they incorporate model selection uncertainty [17] (see methods for details).

### USA

**Figure 3:**
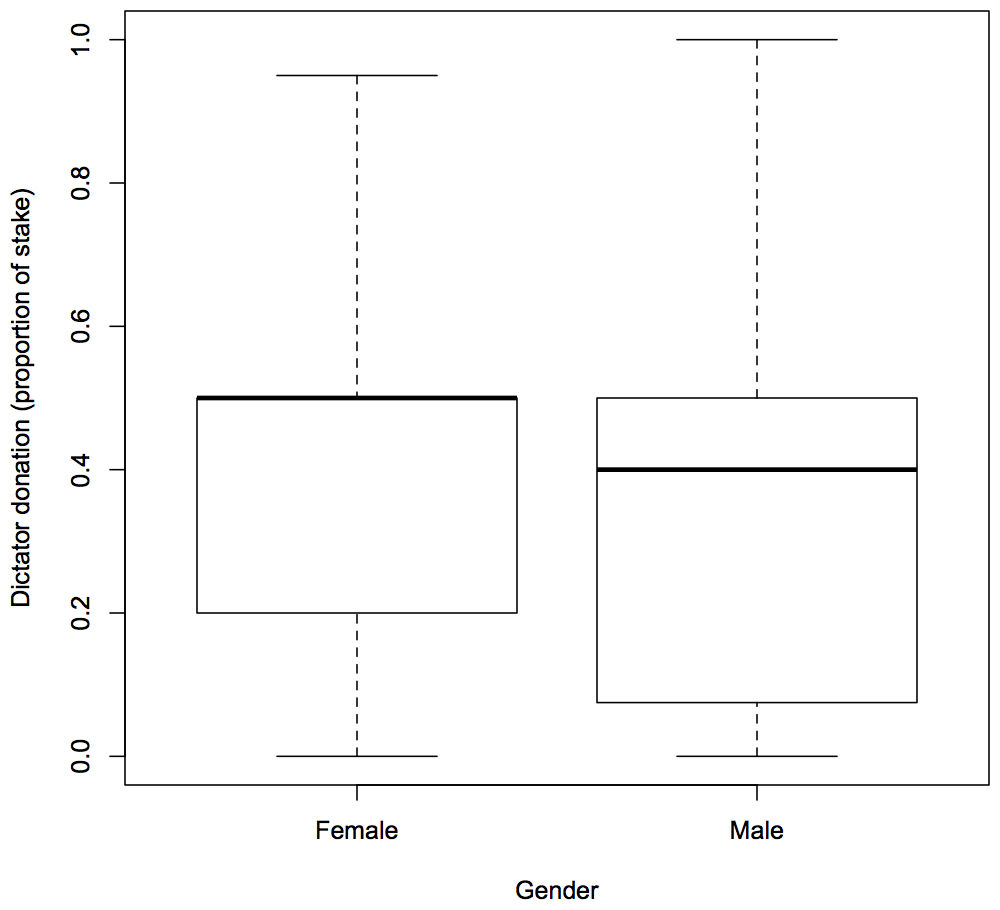
Boxplot of dictator donations (proportion of endowment given to receiver) according to gender for US players. Solid lines represent medians while the upper and lower boundaries of the box are the upper and lower quartiles of the data. The highest and lowest values in the data (excluding outliers) are indicated by the bars extending from the boxes.

For subjects from the USA, we found two models which were within 2 AICc units of the best model (Table 2). Female dictators were more generous than male dictators (importance = 1, Figure 3, Table 4). Stake size was included as a component in the second best model with an importance of 0.3, although, unlike the data from Indian players, there was no clear directional effect of larger stake sizes on dictator donations, and the confidence intervals for all levels of this variable spanned zero (Table 4). Age was also included as a component of the third best model (importance = 0.19) but, as with stake size, the confidence intervals include zero for this term. None of our top models included the year in which data were collected or whether the dictator had children.

## Discussion

This study shows that stake size may affect cooperative behaviour in a Dictator Game. Specifically, the Indian participants were significantly less generous when playing with a $10 stake than with either the $1 or the $5 stake. In contrast, stake size did not seem to affect the behaviour of US participants. We also found that Indian participants were less generous in the second year of the study. Again, this result was not replicated for US participants. Below we discuss some of the possible interpretations of these findings.

**Table 4:** Estimates, unconditional standard errors, confidence intervals and relative importance for parameters included in the top models for players from USA.

**Parameter**	**Estimate**	**Unconditional SE**	**Confidence Interval**	**Relative Importance**
Intercept	0.56	0.03	(0.50, 0.61)	
Gender (F / M)	-0.09	0.04	(-0.17, -0.01)	1.00
Stake				0.30
$1	0.00	0.00	(0.00, 0.00)	
$5	-0.08	0.05	(-0.17, 0.02)	
$10	-0.01	0.05	(-0.10, 0.08)	
Age	-0.01	0.04	(-0.09, 0.07)	0.19

Effect sizes have been standardized on two SD following [18] and standard errors are unconditional, meaning that they incorporate model selection uncertainty [17] (see methods for details).

It is unclear why the Indian players seemed to be affected by the highest stake size in this study. It is likely that stakes of all sizes were more valuable in India - Indian workers report lower median annual income than US workers in AMT (N. Raihani, unpublished data). It may be the case that the $10 stake was very salient for the Indian subgroup; whereas for US participants this stake size was still relatively meaningless. If this were the case, and if subjects generally become less generous as the perceived value of the stake increases, then we might also expect to replicate the negative effect of stake size on generosity in the US subgroup if the 'high stake' treatment were increased to $50 or $100 (although Carpenter et al. 2005 [10] found no effect of increasing stake from $10 to $100 on dictator behaviour using US students). The effect of substantially increasing the stake for US players could be explored in future studies. Another possibility for the apparent sensitivity of Indian players to stake size is that these players might have used AMT chat forums or discussion boards to talk about the game structure and to advise fellow workers that tasks with different stake amounts were available. Although workers are supposed to accept tasks in numerical order, workers are also able to preview tasks in a batch before choosing which one to accept. Arguably, players that 'cheat' in this way may also have been more likely to keep an unfair share of the endowment. We have no way to test whether players were preferentially selecting the highest paying tasks based on information received from discussion boards. We conducted an informal survey which hints that US players are more likely to use discussion boards than Indian players (45/50 US respondents use discussion boards compared with 15/50 Indian respondents, N. Raihani, unpublished data). Moreover, the main discussion board used by AMT workers, MTurk Forum (www.mturkforum.com), does not allow contributors to post information about the content of tasks. Nevertheless, the possibility that players might discuss task content on discussion boards is something for us - and other researchers using AMT - to be aware of for future studies.

We found that the behaviour of the Indian players in this online game varied across years - players were less generous in the second year of the study than in the first year. It is currently not clear why this was the case. The possibility that workers discussed the game in online forums could explain the differences in behaviour between year one and two of the study - again, we do not know whether this is the case but it is possible. Although we are not able to offer a good explanation for the variation in behaviour across year one and two of the study in the Indian players, the fact that we found a temporal difference in generosity indicates that researchers should be cautious when using the 'snapshot' approach to data collection. It may often be the case that patterns of behaviour change temporally but since most studies fail to be replicated over time, such variation may often be obscured.

In this study, male participants from the USA were less generous than females. This finding is consistent with previous Dictator Game studies [23,24] and with a recent meta-analysis of Dictator Games which showed that women on average give significantly more of the initial endowment away than men [8]. The finding that women are more cooperative than men has also been shown in other economic games (e.g. [25,26]) but the effect is not universal [27-29] even in Dictator Games [30]. It has been argued that women may behave more cooperatively than men in such economic games because women are generally more socially-oriented while men are more individually-oriented [23,31], although the ultimate reasons for these proposed differences in male and female preferences are currently unclear. We did not replicate the gender effect among our Indian subgroup, however. Currently, we are not sure why we would find a gender effect on dictator donations among US players but not among Indian players. It is possible that players’ interpretation of the game, or their perceptions of what constitutes a fair donation, may have varied cross-culturally. Indeed, cross-cultural differences have been highlighted in other pro-social and anti-social tendencies previously [3]. To test this hypothesis, we would need to collect data from more countries. However, as described above, our mixed findings with regards to gender differences in economic games are not atypical.

To summarise, online labour markets offer researchers interested in human behaviour a relatively cheap and fast way to generate large bodies of data. Even more importantly, recruiting participants from online labour markets allows researchers to circumvent several of the criticisms that have been levelled against studies using Western undergraduates as the subject base. While there is still a debate to be had about the extent to which AMT workers are truly representative of their particular country or culture, it is clear that there is significant demographic variation among AMT workers that is not present among participants of most laboratory studies. Several studies have been performed which validate the results obtained using AMT against those obtained in laboratory environments. Here, we have shown that varying stake size from $1 to $10 did not have a significant effect on decisions made by US players but that increasing stake sizes seemed to result in decreased generosity among the Indian players. Previous studies have shown that behaviour in economic games can vary across populations [2,3] but our findings raise the possibility that effects of stake size may also show inter-population variation. This is something to be aware of for future studies.

## Supporting Information

Electronic Supplementary Information S1 (DOC)
