# Supplementary material for: The Effect of $1, $5 and $10 Stakes in an Online Dictator Game
Source: PLoS One. 2013 Aug 12;8(8):e73131. doi: 10.1371/journal.pone.0073131 (PMC3741194; doi:10.1371/journal.pone.0073131)
Supplement: Electronic Supplementary Information S1 — (DOC) [file pone.0073131.s001.doc]

**Electronic Supplementary Material:**

**The effect of $1, $5 and $10 stakes in an online Dictator Game**

Nichola J Raihani, Ruth Mace & Shakti Lamba

Subjects were asked to provide information on various demographic variables as below. Not all the information in the questionnaire was collected for this study, hence not all these demographic variables were used in the analyses.

Sample questionnaire and instructions given to subjects

1. What is your sex?

Male / Female

2. What is your age?

3. Which of the following best describes your highest achieved education level?

School up to age 16 / School up to age 18 / Some university no degree / University degree / Graduate degree (Masters / Doctorate etc)

4. Are you currently a student?

Yes / No

5. Are you currently employed in paid work (other than Mechanical Turk)?

Yes / No

6. What is the total income of your household?

Less than $12,500 / $12,500 - $24,999 / $25,000 – $37,499 / $37,500 - $49,999 / $50,000 - $62,499 / $62,500 - $74,999 / $75,000 - $87,499 / $87,500 or more

7. How many people live in your household (including you)?

8. What country did you live in from childhood to the age of 18? Please list all if there were multiple countries.

9. What country do you live in now? Please answer, even if the same as Q8.

10. How long have you lived in that country (years)?

11. Which town / city do you live in now?

12. What is your marital status?

Single / In a relationship, not married / Married / Divorced, single / Divorced, in a relationship

13. How many children do you have? Please type '0' if none.

# The game rules

### You are about to play a game with another worker.

### For this game you have been randomly paired with another worker. One of you will be Player A and the other will be Player B.

### The other player will never know your worker ID and you will not know the other player's worker ID.

### Both players receive $0.20 for successful completion of the HIT. In addition, each player could receive a bonus depending on the decisions made in the game.

### Player A begins with the bonus amount of $1. Player A can choose how much of this bonus money each of the two players will receive by transferring some, none or all of the money to Player B.

### Player A can transfer any amount of the $1 to Player B. Player A keeps the rest of the bonus.

### Here are some examples of the game. The examples will be followed by some questions.

### You must answer the questions correctly to ensure your HIT is accepted.

### Example 1: Player A transfers $0.5 to Player B.

### Both players get $0.5 as a bonus.

### Example 2: Player A transfers $0.8 to Player B.

### Player A gets $0.2 bonus, Player B gets $0.8 bonus.

### Example 3: Player A transfers $0.2 to Player B.

### Player A gets $0.8 bonus,  Player B gets $0.2 bonus.

### Please answer the following questions to ensure you have understood the game.

### If you get the answers wrong then your HIT will be rejected.

1. If Player A transfers $0.1 to Player B, what bonus will Player A get?

|  | $0.10 |
| --- | --- |
|  | $0.90 |

2. If Player A transfers $0.9 to Player B, what bonus will Player B get?

|  | $0.90 |
| --- | --- |
|  | $0.10 |

# Now it's time to play the game!

# You are Player A. Your partner is Player B.

### Player A: you have been allocated a $1 bonus. You can transfer any amount of the $1 bonus to Player B. Your worker ID will not be revealed to the other player.

### Player B: Your bonus will depend on how much of the $1 bonus Player A transfers to you. Your worker ID will not be revealed to the other player.

### Player A: please record your decisions in the boxes below. Please ensure the two amounts sum to $1 to ensure your HIT is accepted.

### Player B: you don't need to fill out these boxes - please leave them blank.

Player A: I will transfer the following amount to Player B: $ ______

This means that I will get $ _____ as a bonus.
